# Pathogenesis and Treatment of Non-alcoholic Steatohepatitis and Its Fibrosis: A Systematic Review

**DOI:** 10.7759/cureus.89969

**Published:** 2025-08-13

**Authors:** Talha Ahmed, Sana Farooq, Muhammad Ashir Naveed, Ali Shahzad, Waleed Rehman, Tauqeer Haider, Naqibullah N Noor

**Affiliations:** 1 Trauma and Orthopaedics, University Hospitals Dorset, Poole Hospital, Poole, GBR; 2 Medicine, HBS Medical and Dental Hospital, Islamabad, PAK; 3 Medical Education, Rashid Latif Medical College, Lahore, Lahore, PAK; 4 General Medicine, Medical and Health Center, University of Agriculture Faisalabad, Faisalabad, PAK; 5 Geriatrics, Barnet Hospital, London, GBR; 6 Nephrology, Allied Hospital, Faisalabad, Faisalabad, PAK; 7 Internal Medicine and Gastroenterology, Amiri Medical Complex, Kabul, AFG

**Keywords:** bariatric surgery, glp-1 agonists, metabolic dysfunction-associated steatohepatitis (mash), metabolic therapy, ppar agonists

## Abstract

Metabolic dysfunction-associated steatohepatitis (MASH), formerly known as non-alcoholic steatohepatitis (NASH), is a slowly progressive liver disease characterized by hepatic steatosis, inflammation, and fibrosis. Despite multiple therapeutic approaches under investigation, no globally approved standard pharmacotherapy currently exists. This systematic review aims to inform and enhance critical care and hepatology practice by synthesizing the most recent evidence on the pathogenesis and treatment of MASH and associated fibrosis. The review was conducted in accordance with Preferred Reporting Items for Systematic Reviews and Meta-Analyses (PRISMA) guidelines. A comprehensive literature search was performed using both text words and controlled vocabulary, incorporating Boolean operators ("AND," "OR") across PubMed, Embase, and the Cochrane Library. The inclusion criteria encompassed open-access, full-text English-language randomized controlled trials (RCTs) published between 2014 and 2024. Study quality was assessed using the RoB 2.0 tool, and the strength of evidence was evaluated using the GRADE framework. Fourteen RCTs were included. Of these, two were rated as high risk of bias (RoB) and consequently downgraded to “low-quality” evidence. Two RCTs with low RoB were classified as “high-quality” evidence, while the remaining 10 trials had unclear RoB, leading to a “moderate-quality” rating due to imprecision. This review discusses clinical trials evaluating therapies such as GLP-1 receptor agonists, THR-β agonists, pan-PPAR agonists, FGF21 analogues, and bariatric surgery. GLP-1 agonists and resmetirom (a THR-β agonist) demonstrated substantial reductions in liver fat content, while lanifibranor (a pan-PPAR agonist) also significantly improved fibrosis. Bariatric surgery showed MASH resolution in 56-70% of cases, though challenges remain regarding incomplete fibrosis reversal and the lack of long-term outcome data. Future research should prioritize combination therapies, explore novel antifibrotic agents, and investigate genetically based therapeutic strategies to better address the multifactorial nature of MASH.

## Introduction and background

Metabolic dysfunction-associated steatohepatitis (MASH) is an advanced form of metabolic dysfunction-associated fatty liver disease (MAFLD), with the characteristic feature being regularly occurring liver damage through inflammation, liver cell injury and scarring (fibrosis) [1]. Disorders that cause MASH mainly refer to insulin resistance, hepatic fat, and oxidative stress, which lead to liver damage and favor the process of chronic inflammation [2]. In addition, the impairment of immune system control and changes in gut microbiota (gut dysbiosis) are of utmost importance in disease development [3]. The intestinal porosity promoted by the dysbiosis phenomenon may contribute to increased penetration into the portal circulation of endotoxins, such as lipopolysaccharides (LPS), and provoke hepatic inflammation. In the long run, sustained injury to the liver stimulates hepatic stellate cells, resulting in excess production of collagen in a process termed fibrosis. Fibrosis may progress to cirrhosis, leading to liver failure in case it is not treated [4].

As the liver progresses to irreversible damage, the hepatic stellate cells become activated and secrete collagen into the liver, resulting in fibrosis, which, in the case of no treatment, can later degenerate to cirrhosis and liver failure. Recently, worldwide, the number of identified cases showed a remarkable increase of almost 8.5 million cases in 22 countries, and the most significant percentage of MAFLD was in South America and the Middle East and the lowest in single countries in Africa [5]. It is well-known that MAFLD is often coupled with obesity, type 2 diabetes, dyslipidemia, and hypertension, as the latter are the characteristics of the metabolic syndrome. In the MASH patients, 40.76% of them acquired advanced fibrosis with an average progression rate of one fibrosis stage in every 11 years [6]. Moreover, patients with MAFLD had a rate of 0.44 per 1,000 person-years of hepatocellular carcinoma (HCC) and 0.77 liver-specific and 11.77 overall deaths per 1,000 person-years, which indicates its increasingly burdening impact on health [7].

Chronic inflammation, driven by pro-inflammatory cytokines (TNF-alpha and IL-6), contributes to MASH, especially when affected persons are obese and have diabetes and metabolic syndrome [8]. Genetic polymorphisms, particularly in the PNPLA3 and TM6SF2 genes, have been identified as important risk factors for the development and progression of MAFLD and its advanced form, MASH. These variants are associated with increased hepatic fat accumulation, inflammation, and fibrosis [9]. At present, lifestyle modifications such as weight loss, a healthy diet, and increased physical activity are the first-line interventions aimed at reducing hepatic fat and inflammation [10]. However, adherence to these changes is often challenging, and there remains an unmet need for effective pharmacological options. Emerging therapies targeting metabolic, inflammatory, and fibrotic pathways have shown promise. For instance, liraglutide, a glucagon-like peptide-1 (GLP-1) receptor agonist, achieved resolution of MASH in 39% of patients by improving insulin sensitivity and reducing liver fat. Similarly, resmetirom, a selective thyroid hormone receptor-β (THR-β) agonist, significantly reduced hepatic fat content by ~50% and lowered noninvasive fibrosis biomarkers [11]. Currently, several therapeutic agents targeting metabolic and fibrotic pathways have shown promising results in the treatment of MASH. Notably, PPAR agonists (e.g., lanifibranor) and FXR agonists (e.g., obeticholic acid) have demonstrated efficacy in improving hepatic steatosis, inflammation, and fibrosis. Some of these agents have already received regulatory approval in specific regions or are in advanced phases of clinical development, reflecting a significant advancement in the pharmacologic management of MASH [12].

While pharmacologic options such as FXR and PPAR agonists have reached late-stage trials and in some cases conditional approval, long-term safety and efficacy data are still being evaluated. As MASH progresses toward cirrhosis, the risk of liver-related complications and mortality increases substantially. This review integrates molecular insights into the pathogenesis of MASH, highlights recent therapeutic developments, and explores emerging drug candidates and clinical trials, providing a comprehensive framework for future research and targeted treatment strategies.

## Review

Material and method

The Preferred Reporting Items for Systematic Reviews and Meta-Analyses (PRISMA) guidelines [13] and Population, Intervention, Comparison, and Outcome (PICO) method [14] were followed in the conduct of this systematic review, described in Table 1.

**Table 1:** Population, Intervention, Comparison, and Outcome (PICO) framework

Concepts	Text Words	Controlled Vocabulary (MeSH)
Population / Problem	"Metabolic dysfunction-associated steatohepatitis," "MASH," "fatty liver," "MASH fibrosis," "MASLD"	"Fatty Liver [MeSH], "Liver Cirrhosis [MeSH], "Non-alcoholic Fatty Liver Disease [MeSH]
Intervention	"pioglitazone," "semaglutide," "GLP-1 agonist," "lifestyle intervention," "diet," "exercise," "fibrosis inhibitors," "investigational drugs"	"Glucagon-Like Peptide 1 [MeSH], "Diet Therapy [MeSH], "Exercise Therapy [MeSH]
Comparative	"placebo," "control group," "standard care," "usual care," "comparator drug"	"Placebos [MeSH], "Standard of Care [MeSH], "Drug Therapy [MeSH]
Outcomes	"fibrosis improvement," "MASH resolution," "ALT," "AST," "NAS score," "side effects," "adverse events," "biomarker change"	"Treatment Outcome [MeSH], "Biomarkers [MeSH], "Adverse Effects [MeSH]

Research Question

What are the underlying mechanisms involved in the pathogenesis of MASH and its progression to liver fibrosis, and what are the current therapeutic interventions for MASH and its associated fibrosis?

Search Strategy and Search Terms

Searches were performed in PubMed using both free-text terms and MeSH terms, such as "Metabolic dysfunction-associated fatty liver disease," "Liver Fibrosis," "Drug Therapy," and "Pathogenesis" Text words and controlled vocabulary using Boolean operators "AND," "OR," and various combinations on PubMed, Embase, and Cochrane Limiters were used to search open-access, full-text, English-language papers related to people from 2014 to 2024.

Search String

The search string included: ("Pioglitazone" OR "Semaglutide" OR "Liraglutide" OR "GLP-1 agonist" OR "Lifestyle modification" OR "Diet therapy" OR "Exercise therapy") AND ("Metabolic dysfunction-associated steatohepatitis" OR "MASH" OR "Metabolic dysfunction-associated fatty liver disease" OR "MAFLD" OR "Pathogenesis") AND ("Liver fibrosis" OR "Hepatic fibrosis" OR "Fibrosis progression") AND ("Treatment outcome" OR "Fibrosis improvement" OR "MASH resolution" OR "Adverse effects" OR "Histological improvement" OR "Biomarkers" OR "ALT" OR "AST").

Inclusion Criteria

This review included only randomized controlled trials (RCTs) involving adult patients of any gender diagnosed with MASH and MASH-related fibrosis. Eligible studies addressed either the pathogenesis or therapeutic interventions for MASH. Only open-access, English-language, full-text articles published between 2014 and 2024 were included.

Exclusion Criteria

The review excluded all other study designs, such as cohort, case-control, observational studies, case reports, case series, conference abstracts, editorials, letters, review papers, and meta-analyses. Studies on teenagers, children, and animals were also excluded. Studies in which outcomes were not related to the pathogenesis and therapeutic advancement of MASH before 2014 were excluded due to restricted data access, incomplete analysis, and payment issues.

Study Selection Process

The initial screening involved two independent reviewers reading the articles' titles and abstracts. Then, the two independent reviewers conducted a full-text review by comprehensively reading the articles. Regarding reviewers' disagreement, a consensus was developed [15]. The review included only those studies that were available in full text and met the inclusion criteria.

Methodological Quality Assessment

The risk of bias was assessed using the Cochrane Risk of Bias 2.0 tool, which divided studies into three categories: high, low, and some concerning risk of bias [16]. The Grading of Recommendations Assessment, Development and Evaluation (GRADE) method was employed to grade trial results into high-quality, moderate-quality, low-quality, and very low-quality [16].

Data Extraction and Synthesis

A datasheet was created to collect details about the data that we need to extract from the included studies for the synthesis of study findings. In terms of the current study, it encompasses basic information, such as Author/Year, Study Design/Population characteristics, Intervention and Dosing, Therapeutic Mechanism, Outcome, Clinical Implication, Safety and Tolerability. After that, a thematic analysis using an inductive, data-driven approach was employed to analyze the data sheet [17]. Then, an iterative approach was applied for a further in-depth study and convergence of the results [18]. Then, studies are analyzed critically to synthesize the evidence, ensuring the practice is evidence-based.

Ethical Consideration

**Figure 1:**
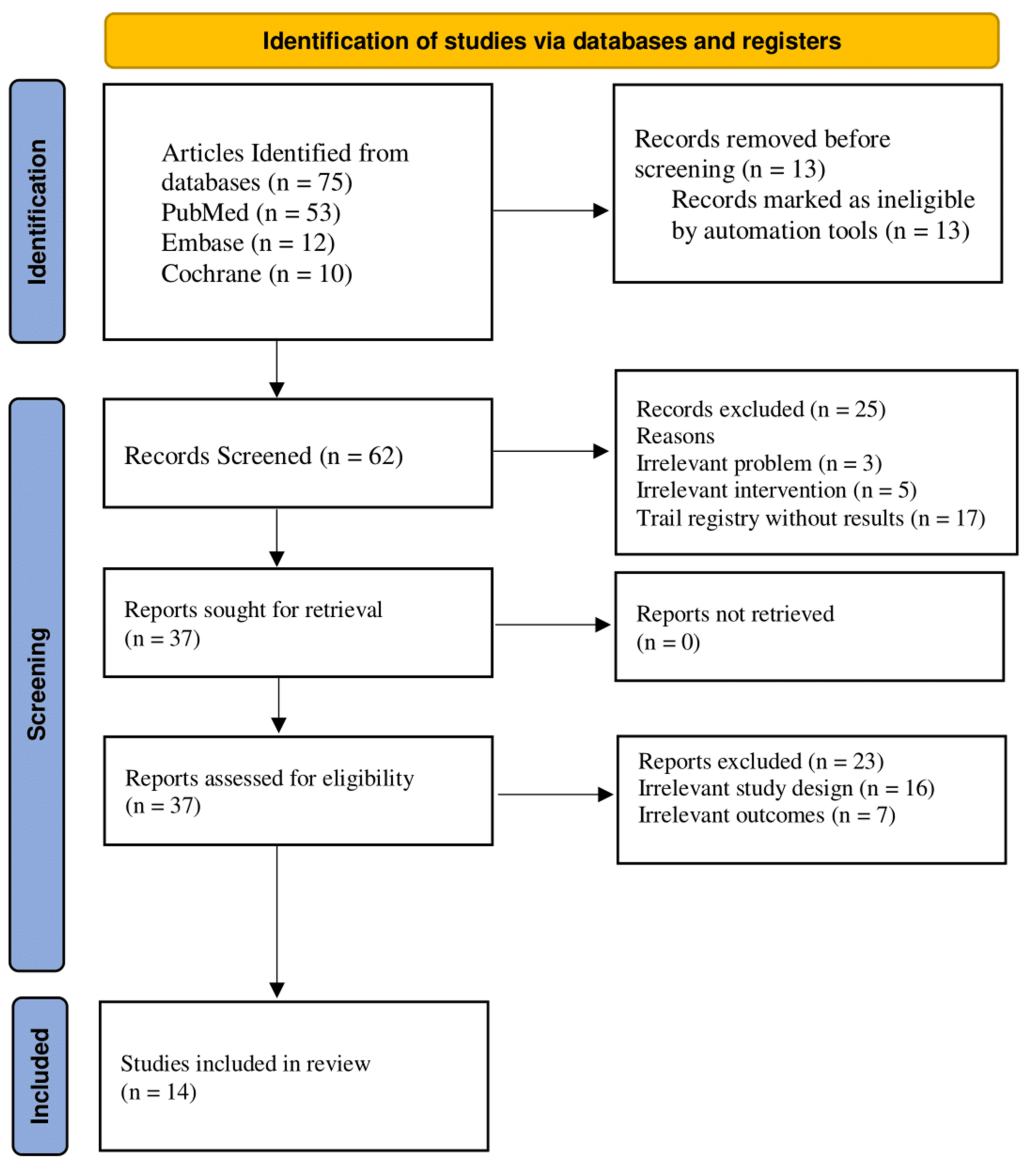
Preferred Reporting Items for Systematic Reviews and Meta-Analyses (PRISMA) Flowchart

The review was conducted in accordance with the Helsinki Declaration to uphold ethical standards. The finding will be published in a medical journal to promote public dissemination while maintaining confidentiality and participant anonymity. Additionally, the study followed the standardized PRISMA guidlines framework to ensure comprehensive and transparent reporting of the review process (Figure 1).

Results

This review followed PRISMA guidelines to synthesize evidence. Seventy-five articles were retrieved from databases like PubMed, Embase, and Cochrane Library. Thirteen duplicates were removed, and 62 articles were selected for screening. Twenty-five irrelevant articles were removed based on title and abstract reading. Thirty-seven articles were eligible, and 23 irrelevant articles were excluded through a thorough review. After eligibility checks, only 14 articles were selected for quality assessment.

Cochrane Risk of Bias Assessment of Studies Included in the Systematic Review

Table 2 presents the Cochrane Risk of Bias assessment for systematic reviews, evaluating potential biases in key domains like selection, performance, detection, and reporting. 

**Table 2:** Cochrane Risk of Bias Cochrane Risk of Bias 2 (RoB 2) is rated as “Low risk,” “Some concerns,” or “High risk” to determine the overall study quality.

Author/Year	Randomisation Process	Deviations from Intended Intervention	Missing Outcome Data	Outcome Measurement	Selection of Reported Result	Overall Quality (RoB)
Harrison et al. 2019 [19]	Low	Some concerns	Low	Low	Low	Moderate
Armstrong et al. 2016 [20]	Low	Low	Some concerns	Low	Low	Moderate
Newsome et al. 2020 [21]	Low	Some concerns	Low	Some concerns	Low	Moderate
Mohamad Nor et al. 2021 [22]	Low	Low	Low	Low	Some concerns	Moderate
Loomba et al. 2023 [23]	Low	Low	Some concerns	Low	Low	Moderate
Francque et al. 2021 [24]	Low	Some concerns	Low	Some concerns	Low	Moderate
Shimizu et al. 2018 [25]	Some concerns	Some concerns	Low	Some concerns	Some concerns	Poor
Ratziu et al. 2016 [26]	Low	Low	Some concerns	Low	Low	Moderate
Verrastro et al. 2023 [27]	Low	Low	Some concerns	Low	Low	Moderate
Sanyal et al., 2022 [28]	Low	Low	Low	Low	Low	High
Loomba, Sanyal, et al., 2023 [29]	Low	Some concerns	Some concerns	Low	Some concerns	Poor
Harrison et al., 2024 [30]	Low	Low	Low	Low	Low	High
Kessoku et al., 2020 [31]	Low	Some concerns	Low	Low	Some concerns	Moderate
Iwaki et al., 2022 [32]	Low	Low	Some concerns	Low	Low	Moderate

The studies generally showed moderate risk of bias, with a few exceptions. Harrison et al. 2019, Armstrong et al. 2016, Newsome et al. 2020, Mohamad Nor et al. 2021, Loomba et al. 2023, Francque et al. 2021, Ratziu et al., Verrastro et al. 2023, Kessoku et al. 2020, and Iwaki et al. 2022 all demonstrated low risk in most domains but had some concerns mainly regarding deviations from intended intervention, missing outcome data, or selection of reported results, resulting in an overall moderate quality [20-25,27,28,32,33]. Shimizu et al. 2018 and Loomba and Sanyal, et al. 2023 were rated poor due to multiple concerns across several domains [26,30], while Sanyal et al. 2022 and Harrison et al. 2024 were rated high quality, showing low risk of bias in all assessed areas [29,31]. Fourteen RCTs were analyzed, with two having low quality, two high quality, and 10 with moderate quality.

Overview of Included Studies and Their Findings

Table 3 details the study design, population characteristics, intervention and dosing, therapeutic mechanism, outcome, clinical implications, and safety and tolerability of the included studies.

**Table 3:** Characteristics and findings of studies included in the review MASH: metabolic dysfunction-associated steatohepatitis, MAFLD: metabolic dysfunction-associated fatty liver disease, SAF-A: Steatosis, Activity, and Fibrosis – Activity score, BMI: body mass index, MRI-PDFF: magnetic resonance imaging – proton density fat fraction, VCTE: vibration-controlled transient elastography, CAP: controlled attenuation parameter, HVPG: hepatic venous pressure gradient, ALT: alanine aminotransferase, AST: aspartate aminotransferase, GGT: gamma-glutamyl transferase, LDL-C: low-density lipoprotein cholesterol, HDL-C: high-density lipoprotein cholesterol, HbA1c: glycated hemoglobin, HOMA-IR: Homeostatic Model Assessment for Insulin Resistance, THR-β: thyroid hormone receptor beta, GLP-1: glucagon-like peptide-1, SGLT2: sodium-glucose cotransporter-2, PPAR: peroxisome proliferator-activated receptor, FGF21: fibroblast growth factor 21, ASK1: apoptosis signal-regulating kinase 1, LOXL2: lysyl oxidase-like 2, Helz2: helicase with zinc finger 2, PRO-C3: N-terminal type III collagen propeptide, ELF: Enhanced Liver Fibrosis score, α-SMA: alpha-smooth muscle actin, MCP® BCMC®: a proprietary blend of Lactobacillus and Bifidobacterium, EXB: elobixibat, an ileal bile acid transporter (IBAT) inhibitor, CTM: cholestyramine, a bile acid sequestrant

Author/year	Study Design/Population characteristics	Intervention & Dosing	Therapeutic Mechanism	Outcome	Clinical Implication	Safety & Tolerability
Harrison et al. 2019 [19]	Randomised Controlled Trial 125 adults with biopsy-confirmed MASH (fibrosis stages 1–3) and hepatic fat fraction ≥10% (MRI-PDFF).	Resmetirom Group: The initial dose of 80 mg daily was adjusted to 100 mg if the AUC was below 3000 ng·h/mL and 60 mg if it was above 5500 ng·h/mL. Placebo Group: Matching oral placebo once daily	Resmetirom is a liver-selective THR-β agonist that enhances mitochondrial β-oxidation, reduces lipotoxicity, and improves metabolic pathways.	Hepatic fat reduction: Resmetirom showed a significant relative decrease in hepatic fat vs. placebo at week 12 (−32.9% vs. −10.4%, p<0.0001) and week 36 (−37.3% vs. −8.9%, p<0.0001). MASH resolution: 27% of Resmetirom-treated patients vs. 6% placebo (p=0.018). Lipid reduction: Significant decreases in LDL-C (−16.2%), triglycerides (−21.4%), and lipoprotein (a) (−22.7%). Liver enzymes: Reduced ALT (−15.4 U/L, p=0.0019) and AST. Fibrosis biomarkers: Improved PRO-C3 and enhanced liver fibrosis scores	Supports Resmetirom as a promising therapy for MASH with fibrosis, improving hepatic fat, histology, and cardio metabolic risk.	Adverse events (AE): Mostly mild (diarrhoea, nausea). No serious drug-related events. No systemic toxicity: No adverse effects on thyroid, bone, or cardiovascular parameters.
Armstrong et al. 2016) [20]	Randomised Controlled Trial Population: 52 overweight adults (BMI ≥25) with biopsy-confirmed MASH (fibrosis stages 1–4, including cirrhosis).	Liraglutide: Subcutaneous injection, titrated to 1.8 mg/day. Placebo: Matching subcutaneous injection.	GLP-1 analogue: Reduces hepatic steatosis via weight loss, improved insulin sensitivity, and direct hepatocyte effects (↓ lipogenesis, ↑ fatty acid oxidation).	Primary: MASH resolution (ballooning score 0 + inflammation ≤1) without fibrosis worsening: Liraglutide: 39% (9/23) vs. Placebo: 9% (2/22) (*RR 4.3, 95% CI 1.0–17.7; p=0.019*). Secondary: Fibrosis progression: 9% (liraglutide) vs. 36% (placebo) (*p=0.04*). Weight loss: −5.5% (liraglutide) vs. −0.7% (placebo) (*p=0.003*). HbA1c reduction: −5.7 mmol/mol (liraglutide) vs. 0 (placebo) (*p=0.03*).	First GLP-1 trial showing histological MASH resolution. addressing cardiovascular risks. Benefits extend to metabolic parameters (weight, HbA1c),	Common AEs: Gastrointestinal (81% liraglutide vs. 65% placebo; nausea, diarrhoea, constipation). Serious AEs: Rare (8% both groups; none drug-related). No cases of pancreatitis or hepatitis.
Newsome et al. 2020 [21]	Randomised Controlled Trial Population: 320 patients with biopsy-confirmed MASH and liver fibrosis (stages F1-F3).	Intervention: Subcutaneous semaglutide (0.1 mg, 0.2 mg, or 0.4 mg once daily). Control: Placebo. Dosing: Dose escalation every 4 weeks to target dose.	Mechanism: GLP-1 receptor agonist. Effects: Reduces weight, improves insulin resistance, and may reduce liver inflammation and fibrosis indirectly.	Primary Endpoint: MASH resolution (no worsening of fibrosis): 59% (0.4 mg) vs. 17% (placebo), P<0.001. Secondary Endpoint: Improvement in fibrosis stage: 43% (0.4 mg) vs. 33% (placebo), P=0.48 (not significant). Weight Loss: Mean -13% (0.4 mg) vs. -1% (placebo). Biomarkers: Reduced liver enzymes (ALT, AST) and inflammation markers.	Semaglutide significantly improves MASH resolution but not fibrosis stage. Potential therapeutic option for NASH,	Common AEs: Nausea (42%), constipation (22%), vomiting (15%). Serious AEs: Gallbladder disorders (7%), neoplasms (15% vs. 8% placebo).
Mohamad Nor et al. 2021 [22]	Randomized controlled trial; 39 MAFLD patients (Malaysian population, obese majority).	Probiotics (MCP® BCMC® strains: 6 strains of Lactobacillus and Bifidobacterium, 30 billion CFU/day) or placebo for 6 months.	Modulation of gut microbiota, stabilization of mucosal immune function, and intestinal barrier integrity.	No significant improvement in hepatic steatosis, fibrosis, or biochemical markers (ALT, AST, cholesterol, etc.). Stabilization of CD8+ T lymphocytes and ZO-1 expression in probiotics group (vs. reduction in placebo).	Probiotics may stabilize intestinal immune function and barrier but lack significant clinical impact on MAFLD progression.	No reported adverse effects; well-tolerated.
Loomba et al. 2023 [23]	Randomized controlled trial; 81 adults with biopsy-confirmed MASH (F1-F3 fibrosis) or phenotypic MASH (high-risk MAFLD).	Intervention: Subcutaneous pegozafermin (3, 9, 18, or 27 mg once weekly; 18 or 36 mg every 2 weeks). Control: Placebo.	Mechanism: GlycoPEGylated FGF21 analogue. Regulates glucose/lipid metabolism, reduces hepatic steatosis, and improves fibrosis biomarkers.	Primary Endpoint: Safety/tolerability confirmed; no serious adverse events. Key Secondary Endpoints: Hepatic fat reduction: Up to -14.9% absolute reduction (27 mg weekly, p<0.0001). ALT reduction: -43.7% (27 mg weekly, p=0.0002). PRO-C3 reduction: -27.7% (27 mg weekly, p=0.0227). Lipid improvements: Reduced triglycerides (-27.6%), LDL-C (-16.5%), and increased HDL-C (+20.1%). Weight loss: -2.18% (27 mg weekly, p=0.038).	Pegozafermin significantly reduces hepatic fat and improves liver/metabolic biomarkers. Potential for less frequent dosing (every 2 weeks).	Common AEs: Increased appetite (16% vs. 0% placebo), diarrhea (13% vs. 22% placebo), headache (11% vs. 6% placebo). Immunogenicity: Anti-drug antibodies in 65% of participants (no neutralization).
Francque et al. 2021 [24]	Randomized controlled trial; 247 patients with biopsy-proven noncirrhotic MASH (76% with significant/advanced fibrosis.	Lanifibranor (pan-PPAR agonist) at 800 mg or 1200 mg once daily vs. placebo for 24 weeks.	Pan-PPAR agonism modulates metabolic, inflammatory, and fibrogenic pathways in NASH.	Primary endpoint: 55% of 1200 mg group achieved ≥2-point SAF-A score reduction (vs. 33% placebo, *P*=0.007). Secondary endpoints: Resolution of MASH (49% vs. 22%), fibrosis improvement (48% vs. 29%), and composite MASH resolution + fibrosis improvement (35% vs. 9%). - Improved ALT, AST, HbA1c, and lipid profiles.	Lanifibranor significantly improves MASH activity and fibrosis	Common AEs: Diarrhea, nausea, peripheral edema, anemia, weight gain (dose-dependent).
Shimizu et al. 2018 [25]	Randomized controlled trial. 57 patients with type 2 diabetes and MAFLD. Mean age: ~56 years; 60% male; Japanese cohort.	Dapagliflozin: 5 mg/day for 24 weeks. Control group: Standard therapy (no SGLT2 inhibitors).	Dapagliflozin is an SGLT2 inhibitor that promotes urinary glucose excretion, reducing hyperglycemia and improving metabolic parameters.	Hepatic steatosis (CAP): Significant reduction in dapagliflozin group (314 → 290 dB/m, P = 0.0424). Liver fibrosis (LSM): Significant reduction in patients with baseline LSM ≥8.0 kPa (14.7 → 11.0 kPa, P = 0.0158). Improved liver enzymes (ALT, AST, GGT) and metabolic markers (HbA1c, HOMA-IR).	Dapagliflozin improves hepatic steatosis and may attenuate fibrosis in patients with MAFLD, particularly in those with significant baseline fibrosis.	Common adverse events: None specifically reported in the study. Serious adverse events: 1 case of congestive heart failure in the control group. No hepatotoxicity or bone density issues observed.
Ratziu et al. 2016 [26]	Randomized controlled trial. 276 patients with non-cirrhotic NASH	Elafibranor 80 mg/day (n=93) Elafibranor 120 mg/day (n=91) Placebo (n=92) Duration: 52 weeks	Dual PPAR-α/δ agonist improves insulin sensitivity, glucose homeostasis, lipid metabolism, and reduces inflammation	Primary outcome (modified definition): MASH resolution without fibrosis worsening (19% vs. 12% placebo; OR=2.31, p=0.045) Post-hoc analysis (NAS ≥4): MASH resolution (20% vs. 11% placebo; OR=3.16, p=0.018) Improved liver enzymes, lipids, and metabolic markers	Elafibranor 120 mg may benefit patients with moderate/severe MASH (NAS ≥4) Potential to improve cardio metabolic risk profile	Mild, reversible increase in serum creatinine (no renal dysfunction)
Verrastro et al. 2023 [27]	Randomized controlled trial. 288 participants with obesity (BMI 30–55 kg/m²),	Interventions: 1. Lifestyle modification + best medical care (n=96). 2. Roux-en-Y gastric bypass (n=96). 3. Sleeve gastrectomy (n=96). Duration: 1-year follow-up.	Lifestyle Modification: Weight loss through diet and exercise. Bariatric Surgery: - Roux-en-Y gastric bypass: Alters gut hormones and reduces nutrient absorption. Sleeve gastrectomy: Reduces stomach size and affects hunger hormones.	Primary Endpoint (NASH resolution without worsening fibrosis): Roux-en-Y gastric bypass: 56% (ITT), 70% (per protocol). Sleeve gastrectomy: 57% (ITT), 70% (per protocol). Lifestyle modification: 16% (ITT), 19% (per protocol). Secondary Endpoint (Fibrosis improvement ≥1 stage): Roux-en-Y gastric bypass: 37% (ITT), 46% (per protocol). Sleeve gastrectomy: 39% (ITT), 47% (per protocol). Lifestyle modification: 23% (ITT), 28% (per protocol). Other Outcomes: Greater weight loss, improved glycaemic control, and lipid profile in surgical groups.	Bariatric-metabolic surgery (Roux-en-Y gastric bypass and sleeve gastrectomy) is significantly more effective than lifestyle modification and medical therapy for resolving MASH and improving fibrosis.	Adverse Events: Severe events: 6% in surgical groups (no re-operations required).
						Common complications: Gastro-oesophageal reflux (19% after sleeve gastrectomy), dumping syndrome, constipation, and minor surgical complications. No deaths or life-threatening
Sanyal et al., 2022 [28]	Randomized controlled trial. 1,135 patients with compensated cirrhosis	1. Simtuzumab (200 mg or 700 mg IV every 2 weeks). 2. Selonsertib (18 mg or 6 mg orally once daily). 3. Placebo. Duration: Median follow-up of 16.6 months.	Simtuzumab: Monoclonal antibody targeting lysyl oxidase-like 2 (LOXL2), an enzyme involved in fibrosis. Selonsertib: Apoptosis signal-regulating kinase 1 (ASK1) inhibitor, reducing inflammation and fibrosis.	Primary Outcome (Cirrhosis Regression): 16% (176/1,135) of patients showed cirrhosis regression (≥1-stage improvement in MASH CRN fibrosis stage). - Regression associated with 6-fold lower risk of liver-related events (HR 0.16; 95% CI 0.04–0.65). Secondary Outcomes: Improvements in non-invasive tests (NITs): ELF score, liver stiffness by VCTE, hepatic collagen, and α-SMA expression. Reduction in HVPG (portal pressure) in regressors.	Cirrhosis regression in MASH is achievable and associated with significantly reduced risk of liver-related complications. Supports the use of histologic regression and NITs as surrogate endpoints in clinical trials.	Adverse Events: No specific safety concerns reported for cirrhosis regression. Simtuzumab and selonsertib were well-tolerated but ineffective in original trials. Liver biopsy-related adverse events were minor and similar across groups. Low incidence of HCC (<1%) observed.
Loomba et al., 2023 [29]	Randomized, -controlled trial. 222 patients with biopsy-confirmed non-cirrhotic MASH (F2/F3 fibrosis, NAS ≥4)	Pegozafermin: 15 mg weekly (n=21) 30 mg weekly (n=73) 44 mg every 2 weeks (n=57) Placebo (n=71, pooled)	Long-acting glycopegylated FGF21 analogue Regulates lipid/glucose metabolism, energy expenditure Reduces hepatic steatosis, inflammation, and fibrosis	Primary Endpoints (vs. placebo): Fibrosis improvement (≥1 stage): 26% (30 mg, P=0.009), 27% (44 mg, P=0.008) MASH resolution: 23% (30 mg), 26% (44 mg) Secondary: Liver fat reduction (MRI-PDFF): -48.2% (30 mg), -41.9% (44 mg) ALT normalization: 59–65% (pegozafermin) vs. 24% (placebo) Improved lipids (↓triglycerides, ↑HDL) and metabolic markers	First FGF21 analogue to show significant fibrosis improvement in NASH Potential for biweekly dosing (convenience) Benefits both liver histology and cardiometabolic risk	Common AEs: Nausea (19–32%), diarrhea (14–24%), injection-site erythema Serious AEs: 4–11% (vs. 4% placebo) Single case of pancreatitis (44 mg group)
Harrison et al., 2024 [30]	Randomized controlled trial. 966 adults with biopsy-confirmed MASH and fibrosis stages F1B, F2, or F3	Intervention: Resmetirom (80 mg or 100 mg once daily, oral) Control: Placebo Duration: 52 weeks (+ planned 54-month follow-up for clinical outcomes)	THR-β-selective agonist Restores mitochondrial function and fatty acid oxidation in the liver Reduces fibrosis and improves lipid metabolism	Primary Endpoints (Week 52): MASH resolution + no fibrosis worsening: 25.9% (80 mg) and 29.9% (100 mg) vs. 9.7% placebo (both *P*<0.001) Fibrosis improvement (≥1 stage) + no MAFLD activity score worsening: 24.2% (80 mg) and 25.9% (100 mg) vs. 14.2% placebo (both *P*<0.001) Secondary: Reduced LDL cholesterol (−13.6% to −16.3% vs. +0.1%; *P*<0.001) Improved liver enzymes (ALT, AST, GGT) and non-invasive markers (MRI-PDFF, FibroScan CAP) No significant weight changes	Resmetirom is the first drug to meet both FDA-recommended histologic endpoints for MASH (fibrosis improvement and MASH resolution) Benefits extend to cardio metabolic risk reduction (LDL, triglycerides)	Adverse Events: 91.6–91.9% resmetirom vs. 92.8% placebo (mostly mild/moderate) Common AEs: Diarrhea (27–33% vs. 16%), nausea (18–22% vs. 13%), pruritus (8–12% vs. 7%) Serious AEs: 10.9–12.7% vs. 11.5% (no drug-induced liver injury)
Kessoku et al., 2020 [31]	Randomized controlled trial. - 100 patients with MAFLD (LDL-C >120 mg/dL, liver fat ≥8% by MRI-PDFF)	Elobixibat (EXB) + Cholestyramine (CTM): 10 mg EXB + 9 g CTM daily (n=25) EXB monotherapy: 10 mg daily (n=25) CTM monotherapy: 9 g daily (n=25) Placebo (n=25)	EXB: Ileal bile acid transporter (IBAT) inhibitor → reduces bile acid (BA) reabsorption, stimulates hepatic BA synthesis from cholesterol CTM: Bile acid sequestrant → binds intestinal BAs, promotes cholesterol catabolism to BAs Combination: Targets hepatic cholesterol accumulation, reduces BA-induced diarrhea	Primary endpoint: Absolute change in LDL-C at 16 weeks (not yet reported) Exploratory endpoints: Liver fat fraction (MRI-PDFF) Hepatic fibrosis (MR elastography) Lipid profiles, liver enzymes, metabolic markers	First trial testing EXB+CTM for MAFLD Potential to address cholesterol-driven MAFLD pathogenesis Non-invasive MRI endpoints may reduce need for liver biopsy	Expected AEs (EXB): Diarrhea, abdominal pain CTM: May mitigate EXB-induced diarrhea
Iwaki et al., 2022 [32]	Randomized controlled trial. 28 adult patients with MAFLD/NASH	Intervention: Guanabenz acetate (4 mg or 8 mg daily, orally, for 16 weeks). Dosing: Two groups (n=14 each) receiving 4 mg or 8 mg.	Binds to helicase with zinc finger 2 (Helz2), inhibiting hepatic lipid accumulation. Reduces lipogenesis (Scd1 inhibition) and enhances fatty acid β-oxidation (Cpt1a activation). Improves insulin resistance and hyperlipidemia.	Primary: Reduction in hepatic fat content (MRI-PDFF) by ≥3.46% at 16 weeks. Secondary: Changes in liver enzymes, weight, blood lipids, HOMA-IR, fibrosis markers.	Potential dual benefit for MAFLD and metabolic comorbidities (e.g., hypertension, insulin resistance). Targets Helz2, a novel mechanism for metabolic liver diseases.	AEs/SAEs monitored, including hypotension, sedation (central α2-agonist effects). Prohibited concomitant drugs: CNS depressants, hepatotoxins.

MASH is a progressive form of MAFLD, characterised by hepatic steatosis (fat accumulation), inflammation, and hepatocellular injury. If left untreated, this condition can progress to liver fibrosis, cirrhosis, and ultimately liver failure. The elucidation of the pathogenic patterns also plays a significant role in order to work out profitable treatment programs.

Metabolic Dysfunction and Lipotoxicity

Diehl and Day and Friedman et al. claim that the pathogenesis of MASH and its evolution to advanced fibrosis can be considered a multifactorial and complex process. It has mainly been facilitated by a combination of metabolic dysfunction, hepatocellular damage, oxidative stability, immune caravan movement, and heredity [33,34]. Excessive buildup of free fatty acids as well as the toxic intermediates of lipid metabolism result in lipotoxicity that leads to mitochondrial dysfunction and endoplasmic reticulum (ER) stress, which causes cell damage. Exposure of hepatocytes to any damaging condition triggers the release of damage-associated molecular patterns (DAMPs) that serve as an activator of the innate response. The consequence of such a response is the activation of intrahepatic liver immune cells, which eventually causes persistent inflammation and a stimulating fibrogenic pathway causing liver tissue scarring to occur [35,36]

Inflammation and Fibrosis Development

According to Machado and Diehl, hepatocellular injury in MASH triggers a sterile inflammatory response that recruits and activates hepatic immune cells, including Kupffer cells, monocyte-derived macrophages, and neutrophils [37]. These immune cells release pro-inflammatory cytokines such as tumor necrosis factor-alpha (TNF-α), interleukin-1 beta (IL-1β), and interleukin-6 (IL-6), which amplify and sustain hepatic inflammation. In parallel, hepatic stellate cells (HSCs) are activated and transdifferentiate into myofibroblast-like cells, as noted by Schwabe et al. These cells are primarily responsible for the pathological deposition of extracellular matrix (ECM) components, particularly collagen types I and III, driving the progression of liver fibrosis [38]. Musso et al. highlighted that emerging non-invasive biomarkers such as PRO-C3, the Enhanced Liver Fibrosis (ELF) score, and cytokeratin-18 fragments offer promising tools for quantifying fibrotic activity and monitoring disease progression [39].

Gut-Liver Axis and Microbiota

Sharma et al. emphasized that the gut-liver axis plays a crucial role in regulating hepatic inflammation in MASH. Intestinal dysbiosis, characterized by an imbalance in microbial populations, and reduced epithelial barrier integrity marked by downregulated expression of tight junction proteins such as zonula occludens-1 (ZO-1) facilitate the translocation of bacterial endotoxins like lipopolysaccharides (LPS) into the portal circulation. These endotoxins activate toll-like receptor 4 (TLR4) signaling pathways in hepatic immune cells, further aggravating liver inflammation. Although probiotic supplementation with strains such as MCP® BCMC® (a proprietary blend of Lactobacillus and Bifidobacterium) has shown potential in improving gut barrier function and reducing systemic inflammation, current clinical studies have not demonstrated significant improvements in liver histology [40].

Genetic Susceptibility and Novel Molecular Targets

Genetic predisposition plays a critical role in the onset and progression of MASH. Sheka et al. identified several gene variants PNPLA3, TM6SF2, MBOAT7, and GCKR that are associated with increased hepatic lipid accumulation, inflammation, and fibrogenesis [41]. These polymorphisms influence both disease severity and response to therapy. Furthermore, Younossi et al. proposed Helz2, a transcriptional co-regulator involved in hepatic lipid metabolism, as a promising molecular target [42]. At the preclinical level, hepatic steatosis was ameliorated and insulin sensitivity enhanced by pharmacologic Helz2 blockade by guanabenz acetate. The stated genetic and molecular insights are a starting point to devise future precision medicine solutions that can curb or reverse the occurrence of MASH.

Treatment of NASH and Its Fibrosis

With the improved understanding of the multifactorial pathophysiology of MASH, approaches to treating it have continued to diversify, targeting a variety of disease processes, including metabolic regulatory and insulin-sensitizing effects to direct antifibrotic activity. Such interventions are pharmacological and non-pharmacological.

Resmetirom (THR-β agonist): The most promising so far selective THR-β agonist is resmetirom, which exerts its effect by increasing hepatic fat burning and mitochondrial functioning, as well as clearing lipid harmful substances. In fact, Armstrong et al. proved that it changed the liver fat substantially and enhanced the profile of liver enzymes and fibrosis indicators, like PRO-C3 [20]. Most recently, it was again established that resmetirom was able to achieve both targeted histological endpoints per the FDA recommendations: resolution of MASH without aggravation of fibrosis and improved fibrosis by at least one stage (Harrison, Slavin, Dell, Soohoo, and Beavers, 2024). It resulted, also, in positive shifts in low-density lipoproteins (LDLs), triglycerides, as well as non-invasive imaging cardinal measures (e.g., magnetic resonance imaging-proton density fat fraction (MRI-PDFF), FibroScan). The drug possesses a favourable safety profile that presents only mild-to-moderate adverse events and no systemic toxicity with respect to thyroid, cardiovascular and skeletal systems [19]. Such repeated results indicate resmetirom as a candidate among the first proposals of MASH treatment.

GLP-1 receptor agonists liraglutide and semaglutide: Liraglutide and semaglutide are examples of GLP-1 receptor agonists which are antidiabetic agents that have been established as effective antidiabetic interventions in MASH owing to their interventional impacts in weight regression, insulin sensitivity, and hepatic metabolism. Armstrong et al. revealed that in placebo-predominant and liraglutide there was a reduction in the resolution of MASH that was shown as 39% in liraglutide versus 9% in placebo, and the decrease in fibrosis progression was also significant [20]. In the same way, there is the study by Newsome et al. which indicates that semaglutide reached the MASH resolution in 59% of the patients involved in the trial, but no statistically improved fibrosis was detected [21]. These agents also corrected triglycerides, liver enzymes and the levels of HbA1c. The most frequently occurring adverse effects were gastrointestinal (as it happened e.g. nausea, constipation) and controllable. The fact that they do have two benefits makes GLP-1 agonists attractive drugs in the treatment of MASH.

Lanifibranor (pan-PPAR agonist): Lanifibranor is a pan-PPAR (alpha, delta, gamma) agonist with the modulation of metabolic, inflammatory and fibrogenic pathways. Francque et al. conducted a randomized controlled trial where 55% of patients who had undergone 1200 mg demonstrated SAF-A score improvement of two points and 49% of the patients had a MASH resolution and 48% showed improvement in fibrosis. Particularly, 35% of interviewees managed to meet both extremes. There was also a good decrease in alanine aminotransferase (ALT), aspartate aminotransferase (AST), HbA1c along with the betterment of lipid profiles [24]. These same findings were also corroborated by Loomba et al. (2023). Side effects were gastrointestinal side effects that are dose-dependent, edema, and anemia. In this way, lanifibranor provides a multi-targeted approach to MASH through hepatic and systemic aspects [23].

Pegozafermin (FGF21 analogue): Pegozafermin, a glycopegylated FGF21 analogue, improves lipid and glucose metabolism, energy regulation, and fibrogenic pathways. In trials by Loomba et al. (2023), it reduced hepatic fat content by 41.9% to 48.2%, normalized ALT in up to 65% of patients, and led to fibrosis improvement in 26-27% of cases. Rates of MASH resolution were similarly encouraging [23]. It also improved triglyceride and high-density lipoprotein (HDL) levels, with tolerable side effects including nausea, diarrhea, and injection-site reactions. Its biweekly dosing enhances adherence, and it is the first FGF21 analogue to demonstrate robust histological benefits in MASH [24].

Dapagliflozin (SGLT2 inhibitor): Elafibranor modulates lipid metabolism, glucose regulation, and inflammation. Shimizu et al. demonstrated MASH resolution in 20% of patients receiving 120 mg/day versus 11% with placebo, without fibrosis worsening. Improvements in liver enzymes and lipid parameters were also noted. Mild adverse effects included a transient rise in serum creatinine [25]. Although it is no longer a frontrunner in drug pipelines, elafibranor provided valuable insights into PPAR modulation in MASH [26].

Elafibranor (dual PPAR-α/δ agonist): Elafibranor modulates lipid metabolism, glucose regulation, and inflammation. Ratziu et al. demonstrated MASH resolution in 20% of patients receiving 120 mg/day versus 11% with placebo, without fibrosis worsening. Improvements in liver enzymes and lipid parameters were also noted. Mild adverse effects included a transient rise in serum creatinine [26]. Although it is no longer a frontrunner in drug pipelines, elafibranor provided valuable insights into PPAR modulation in MASH [27].

Selonsertib and simtuzumab (antifibrotic agents): Selonsertib (ASK1 inhibitor) and simtuzumab (LOXL2 inhibitor) were developed to directly halt fibrogenesis. Despite underwhelming results in initial trials, Sanyal et al. reported ≥one-stage cirrhosis regression in 16% of patients, which was associated with a six-fold lower risk of liver-related complications. Improvements in ELF score, hepatic collagen, and HVPG were observed in fibrosis repressor [28]. These agents, while no longer in clinical development, highlighted the feasibility of fibrosis reversal and the importance of surrogate endpoints [29].

Bariatric-metabolic surgery: Bariatric procedures, such as Roux-en-Y gastric bypass and sleeve gastrectomy, have shown dramatic efficacy in MASH. In the BRAVES trial, MASH resolution occurred in 56-70% of surgical patients versus 16-19% in the lifestyle and medical care group. Fibrosis improvement (≥one stage) was achieved in 46-47% of surgical patients. Benefits included significant weight loss, improved glycemic control, and lipid profile enhancement. While mild complications like dumping syndrome and reflux were reported, no serious events occurred [27]. Bariatric surgery remains an effective option in patients with severe MASH and obesity [30].

Lifestyle and diet: Lifestyle interventions remain the cornerstone of MASH management. Weight loss of ≥7-10% via caloric restriction and physical activity is associated with improvements in steatosis, inflammation, and fibrosis. However, long-term adherence remains challenging. In the BRAVES trial, only 16-19% of patients in the lifestyle group achieved MASH resolution [27,31]. Despite these limitations, lifestyle changes are critical for overall cardiometabolic health and may enhance pharmacologic efficacy when used in combination.

Gut microbiota modulation: The gut-liver axis is an emerging therapeutic target. Iwaki et al. evaluated MCP® BCMC® probiotics (strains of Lactobacillus and Bifidobacterium) and found improvements in gut barrier integrity and immune modulation (e.g., increased ZO-1 expression, stabilized CD8+ T cells). However, no significant changes were observed in liver steatosis or fibrosis [32]. Similarly, Kessoku et al. noted that while probiotics may support gut immunity, conclusive evidence for liver benefit remains lacking. Ongoing trials are assessing synbiotics and fecal microbiota transplantation. Gut microbiota modulation remains promising but is not currently recommended as monotherapy for MASH [31].

Discussion

MASH presents a significant therapeutic challenge due to its multifaceted pathophysiology involving metabolic derangements, chronic inflammation, and progressive fibrosis. Recent studies have explored a range of treatment strategies targeting these core mechanisms, with varying degrees of success.

MASH is primarily driven by metabolic dysfunction particularly insulin resistance and lipotoxicity. According to Armstrong et al., liraglutide, a GLP-1 receptor agonist, resulted in histological resolution of MASH in 39% of patients, significantly improving insulin sensitivity and reducing hepatic fat content [20]. Similarly, Zhang et al. demonstrated that resmetirom, a selective THR-β agonist, reduced liver fat by approximately 50% and lowered key fibrosis biomarkers, reinforcing the importance of targeting metabolic pathways in MASH management [43]. Fu et al. reported that lanifibranor, a pan-PPAR agonist, improved lipid metabolism, led to MASH resolution in 49% of patients, and produced ≥1-stage fibrosis improvement in 35% of cases [44].

In general, these findings emphasize that metabolic modulation is foundational in halting disease progression. However, variability in fibrosis response indicates that patients with advanced fibrotic MASH may benefit from adjunct antifibrotic therapies. Inflammation and fibrosis represent additional therapeutic targets. Ratziu et al. showed that elafibranor reduced hepatic inflammation and achieved MASH resolution in a subset of patients, though it had limited impact on fibrosis [26].

Golabi et al. reported that pegozafermin, an FGF21 analogue, modulated inflammation and improved fibrosis in 27% of patients [45]. These findings suggest that agents with broader anti-inflammatory profiles, such as pan-PPAR agonists, may offer superior efficacy in fibrosis attenuation. Musso et al. observed only modest cirrhosis regression (16%) with the antifibrotic agents selonsertib and simtuzumab, highlighting the limitations of monotherapy in addressing advanced fibrosis [46]. Collectively, these studies underscore the need for combination regimens that simultaneously address metabolic, inflammatory, and fibrotic pathways. Beyond pharmacotherapy, the gut-liver axis and surgical interventions have also been explored. Mohamad Nor et al. found that while probiotics (e.g., MCP BCMC strains) modestly improved gut barrier integrity, they did not significantly improve liver histology, supporting their role as adjunctive rather than primary therapies [22].

In contrast, Lu et al. demonstrated that bariatric surgery resolved MASH in 56-70% of patients and improved fibrosis in nearly half, indicating its substantial metabolic benefits [47]. However, surgical interventions are not universally applicable. Lifestyle interventions remain the cornerstone of MASH management but yielded modest success, with MASH resolution achieved in only 16-19% of patients in real-world settings, reflecting challenges in adherence and long-term maintenance [27]. The robust outcomes from resmetirom trials further highlight the potential of agents that target hepatic transcriptional regulation [23]. Given the heterogeneity of MASH, clinicians should consider personalized treatment strategies that address the predominant pathogenic mechanism metabolic dysfunction, inflammation, or fibrosis-based on disease stage. Ongoing research should prioritize combination approaches and the use of predictive biomarkers to guide therapy.

Clinical Implication

The management of MASH has entered a transformative phase with the emergence of targeted therapies. Metabolic agents such as GLP-1 receptor agonists (liraglutide, semaglutide) and THR-β agonists (resmetirom) are now front-line options due to their ability to improve insulin sensitivity, reduce hepatic steatosis, and achieve histologic resolution. Broader-acting agents like pan-PPAR agonists (lanifibranor) and FGF21 analogues (pegozafermin) further extend therapeutic options, particularly in patients with more advanced fibrosis or concurrent inflammatory features. Bariatric surgery remains the most effective intervention for eligible patients with severe obesity and MASH, with resolution rates exceeding 60%. However, accessibility and patient eligibility limit its broader application. While lifestyle modification is universally recommended, its modest efficacy and poor adherence in real-world settings necessitate adjunct pharmacologic or surgical interventions for long-term disease control.

Limitations and Future Direction

Despite therapeutic advancements, several limitations persist. Many agents exhibit variable efficacy in fibrosis reversal, and there is limited long-term data on their impact on cirrhosis progression and liver-related mortality. Lifestyle interventions remain difficult to sustain, and microbiota-based therapies are still experimental. In addition, the absence of validated non-invasive biomarkers and limited availability of genetic profiling impede precision treatment. Future directions should focus on the development of combination therapies targeting multiple pathogenic pathways, the discovery of novel antifibrotic agents, and the validation of non-invasive diagnostic and prognostic tools. Advancing precision medicine approaches through genotyping and biomarker stratification will be essential to tailor treatment to individual disease phenotypes. Longitudinal studies are also needed to evaluate long-term benefits and safety outcomes. Until more definitive treatments emerge, a multidisciplinary approach incorporating lifestyle modification, pharmacotherapy, and, when indicated, metabolic surgery remains the most effective strategy for MASH management.

## Conclusions

MASH is a complex and progressive liver disease driven by interconnected metabolic, inflammatory, and fibrotic mechanisms. Although no single treatment currently offers a cure, substantial progress has been made in developing targeted pharmacologic agents that show promising results in MASH resolution and fibrosis improvement. Bariatric surgery remains highly effective for eligible patients, while lifestyle modification continues to be foundational, albeit challenging to maintain. Moving forward, the integration of combination therapies, personalized medicine, and long-term outcome research will be critical to improving patient outcomes in MASH.
